# Associations of Parity With Change in Global Cognition and Incident Cognitive Impairment in Older Women

**DOI:** 10.3389/fnagi.2022.864128

**Published:** 2022-05-04

**Authors:** Rui Zhou, Hua-Min Liu, Lian-Wu Zou, Hong-Xia Wei, Yi-Ning Huang, Qi Zhong, Shan-Yuan Gu, Ming-Feng Chen, Shao-Li Wang, Hai-Xia Sun, Xian-Bo Wu

**Affiliations:** ^1^Department of Epidemiology, School of Public Health (Guangdong Provincial Key Laboratory of Tropical Disease Research), Southern Medical University, Guangzhou, China; ^2^Department of Psychiatry, Baiyun Jingkang Hospital, Guangzhou, China; ^3^Department of Biostatistics, School of Public Health (Guangdong Provincial Key Laboratory of Tropical Disease Research), Southern Medical University, Guangzhou, China; ^4^Inpatient Department, Baiyun Jingkang Hospital, Guangzhou, China; ^5^Clinical Laboratory, Baiyun Jingkang Hospital, Guangzhou, China; ^6^Department of Geriatrics, The 74th Army Hospital of the Chinese People’s Liberation Army, Guangzhou, China

**Keywords:** cognitive decline, cognitive impairment, dementia, epidemiology, parity

## Abstract

**Background:**

The evidence of the association between parity and risk of mild cognitive impairment (MCI) or dementia is mixed, and the relationship between parity and longitudinal cognitive changes is less clear. We investigated these issues in a large population of older women who were carefully monitored for development of MCI and probable dementia.

**Methods:**

Using the Women’s Health Initiative Memory Study, 7,100 postmenopausal women (mean age 70.1 ± 3.8 years) with information on baseline parity (defined as the number of term pregnancies), measures of global cognition (Modified Mini-Mental State Examination score) from 1996–2007, and cognitive impairment (centrally adjudicated diagnoses of MCI and dementia) from 1996–2016 were included. Multivariable linear mixed-effects models were used to analyze the rate of changes in global cognition. Cox regression models were used to evaluate the risk of MCI/dementia across parity groups.

**Results:**

Over an average of 10.5 years, 465 new cases of MCI/dementia were identified. Compared with nulliparous women, those with a parity of 1–3 and ≥4 had a lower MCI/dementia risk. The HRs were 0.75 (0.56–0.99) and 0.71 (0.53–0.96), respectively (*P* < 0.01). Similarly, a parity of 1–3 and ≥4 was related to slower cognitive decline (β = 0.164, 0.292, respectively, *P* < 0.05).

**Conclusion:**

Higher parity attenuated the future risk for MCI/dementia and slowed the rates of cognitive decline in elderly women. Future studies are needed to determine how parity affects late-life cognitive function in women.

## Introduction

A mounting body of evidence supports that dementia is a disorder with sex differences, and women show a greater prevalence of dementia, especially Alzheimer’s disease (AD), the most common type of dementia, than men (Jia et al., 2020; Guo et al., 2021). Moreover, while males overall appear to be at a slightly higher risk for vascular dementia throughout most of the lifespan, this trend is reversed at advanced ages (age 85) (Gannon et al., 2018). Pregnancy and childbirth, which are the most distinctive experiences of women, were hypothesized to affect late-life cognition through understudied biological pathways and thus contribute to sex differences in dementia risk (Fox et al., 2018; G de Lange et al., 2020). For instance, pregnancy events leave “residual signatures” on inflammatory markers, blood counts, and telomere length that could in turn influence immunologic and inflammatory trajectories over the life course, thereby altering the risk for dementia (Cramer and Vitonis, 2017; Pollack et al., 2018). Pregnancy might also have an impact on women’s brain aging trajectories through modifying estrogen levels that may have direct and indirect effects on neurotransmitters, modulate neuronal structures, enhance synaptic plasticity, increase cerebral blood flow, and increase breakdown of β-amyloid precursors (Genazzani et al., 2007; Naftolin et al., 2018; Peterson and Tom, 2021). It may also be attributable to various pregnancy-associated factors such as health, employment, and lifestyle during or after pregnancy (Jang et al., 2018).

Several studies have investigated the relationship between parity and risk of cognitive impairment and dementia, but existing evidence is far from conclusive and yields mixed findings. In a retrospective study from Singapore on older women, grand multiparous women (five or more childbirths) showed about 1.3-fold higher risk of cognitive impairment than women with 1–2 parities (Xingyue et al., 2020). Another pooled study of six population-based, prospective cohort studies from four European and two Asian countries yielded similar results on all-cause dementia and non-AD dementia, though the relationships were not uniform across regions (Bae et al., 2020). By contrast, a prospective analysis using the American claims database has suggested that women with three children and those with ≥4 children are associated with a lower risk of all-cause dementia compared with women with one child (Gilsanz et al., 2018), whereas others found no association between parity and risk of all-cause dementia or AD (Shimizu et al., 2019; Najar et al., 2020; Gemmill and Weiss, 2021). None of the studies included detailed information on pregnancy, as parity was roughly defined as the number of children, and some studies did not assess hormonal replacement therapy exposure (Gilsanz et al., 2018; Bae et al., 2020; Gemmill and Weiss, 2021). In addition, growing attention needs to be directed to exploring the long-term neurocognitive effects of parity (Levine et al., 2021). Although a cross-sectional study has related higher number of parity to better memory ability in elderly women (Henderson et al., 2003), the long-term effect of parity on changes in cognitive function is much less clear, which needs to be better understood.

Therefore, we more accurately examined the effect of parity, which was defined as the number of pregnancies lasting 6 or more months (including live births and stillbirths) on the risk of incident mild cognitive impairment (MCI) or probable dementia (PD). Furthermore, we quantified the long-term impact of parity on cognitive change in a large, multiethnic, population-based cohort of older women in the United States.

## Materials and Methods

### Study Population

The Women’s Health Initiative Memory Study (WHIMS) (Shumaker et al., 1998) was an ancillary study of the previously published Women’s Health Initiative hormone therapy (WHI-HT) trials (The Women’s Health Initiative Study Group, 1998), which were two large, randomized, double-blind, and placebo-controlled clinical trials of conjugated equine estrogen treatment alone (E-alone) for women with prior hysterectomy or in combination with progestin (E + P) for women with an intact uterus. The goal of WHIMS was to investigate the association between postmenopausal hormone therapy and risk for PD and cognitive decline. The study design, eligibility criteria, and recruitment procedures of the WHI and WHIMS have been reported elsewhere (Shumaker et al., 1998; The Women’s Health Initiative Study Group, 1998).

**FIGURE 1:**
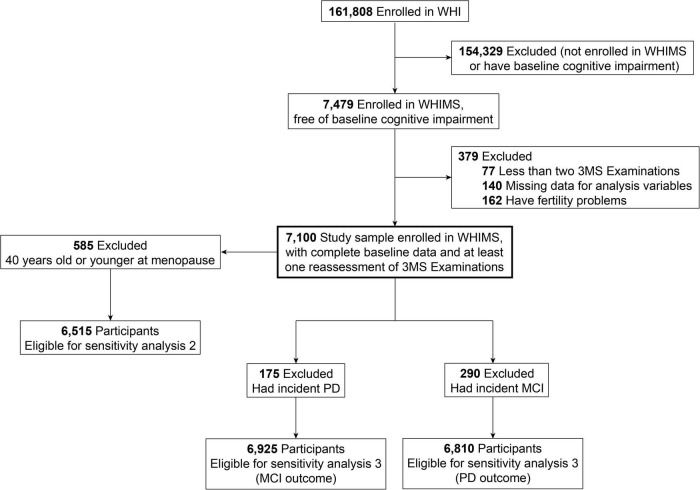
Flow chart.

During 1995–1998, the WHIMS initially recruited 7,479 participants who were 65 years old and older and free of dementia at enrollment (as defined by WHIMS protocols from WHI-HT, see Supplementary Appendix). We excluded women who had missing data on parity (*n* = 140), were told by a doctor to have problems in fertility (*n* = 162), or had no longitudinal data available on cognitive status following enrollment (*n* = 77). Finally, 7,100 women were included in the analyses (Figure 1). Informed written consent was obtained from all participants. The National Institutes of Health and the Institutional Review Boards for the WHI clinical coordinating center and each WHI clinical center approved the study protocols.

### Parity Assessment

Information on parity characteristics of each participant was collected at baseline by self-administered questionnaires. We assigned parity as the number of term pregnancies (i.e., lasting 6 or more months including live births and stillbirths) (Shadyab et al., 2016). Participants were categorized into three groups based on parity: 0 (never been pregnant or had term pregnancies), 1–3, and 4 or more (multiparity) (Gemmill and Weiss, 2021).

### Assessment of Cognitive Function

Cognitive function was assessed at baseline and annually using the Modified Mini-Mental State Exam (3MSE) administered by trained and certified technicians. This approach allowed screening positively for cognitive impairment and tracking the rate of progression of cognitive decline (Teng and Chui, 1987; Espeland et al., 2004). The scores of 3MSE ranged from 0 to 100, and a higher score reflected better cognitive function. The 3MSE tests comprised items within nine dimensions, namely, temporal and spatial orientation, immediate and delayed recall, executive function, naming, verbal fluency, abstract reasoning, praxis, writing, and visuoconstructional abilities (Teng and Chui, 1987).

The cognitive status of each participant was evaluated at annual follow-up assessments. Details on outcome ascertainment and case adjudication have been described previously (Espeland et al., 2004; Shumaker et al., 2004) and included in the Supplementary Appendix. Briefly, during the WHIMS study period (1995–2007), women who scored below a set threshold on the 3MSE underwent an additional cognitive testing and clinical assessment, including a battery of neuropsychologic tests, history and physical, and neuropsychiatric evaluation (Shumaker et al., 1998). From 2008 to 2016, the WHIMS Epidemiology of Cognitive Health Outcomes (WHIMS-ECHO) continued the follow-up of WHIMS via telephone-based assessments. Participants were screened with the Modified Telephone Interview for Cognitive Status (TICS-M) (Welsh et al., 1993), and the transfer from 3MSE assessment to TICS-M was justified in a validation study (Arnold et al., 2009). For women who screened positive (i.e., TICS-M < 31) during the WHIMS-ECHO follow-up, the standardized Dementia Questionnaire (Ellis et al., 1998), a validated instrument that assesses cognitive, behavioral, and functional status, was administered to a reliable and preidentified informant via telephone.

All available data of participants in WHIMS and WHIMS-ECHO were then transmitted to the central adjudication committee. The committee had experts experienced in neurological examinations and neuropsychiatric evaluations, wherein participants were classified as having PD, MCI, or no cognitive impairment based on the *Diagnostic and Statistical Manual of Mental Disorders, Fourth Edition (DSM-IV)* criteria (American Psychiatric Association, 2000). For MCI, the criteria were as follows: (Jia et al., 2020) reported memory problems (Guo et al., 2021), an objective memory deficit measured by cognitive tests (Gannon et al., 2018), normal global cognitive function (G de Lange et al., 2020), absence of significant functional impairment, and (Fox et al., 2018) absence of dementia. The present study included two end points, namely, longitudinal changes in 3MSE scores and a composite outcome that included incident cases of PD or MCI (first event of either outcome).

### Covariates

Using baseline questionnaires, we obtained the following potential confounders: age (continuous variable); race and ethnicity (white and non-white); educational level (high school or less, high school or the Tests of General Educational Development, some school after high school, and college degree or higher); employment status (currently employed or not); family income (<$19,000, $20,000–$34,999, $35,000–$49,999, and ≥$50,000); social support construct scores (continuous); marital status (married or not); age at menopause (continuous variable); body mass index [BMI, calculated as weight (kg)/[height (m)]^2^]; smoking status (never, former, and current); alcohol consumption (non-drinker, past drinker, current and fewer than one drinks per day, and current and one or more drinks per day); physical activity (no activity, some activity, 2–4 episodes per week, and 4 or more episodes per week) (Chen et al., 2015); self-reported medical information on major chronic diseases, including hypertension, diabetes mellitus (DM), and cardiovascular diseases (CVDs); depression symptom; lipid-lowering medication history (yes or no); and hormone therapy treatment. Hypertension was defined as self-reported hypertension history or antihypertensive drug use. DM was defined as self-reported having been diagnosed with diabetes by a doctor plus history of oral medication or insulin therapy. History of CVD included previous coronary heart disease (myocardial infarction, coronary angioplasty, or coronary artery bypass graft), stroke, or transient ischemic attack. The good reliability and validity of the self-reported medical histories and the physical measures have been documented (Langer et al., 2003). Depression was measured using an 8-item screening instrument developed for the Medical Outcomes Study that incorporated 6 items about depressive symptoms from the Center for Epidemiological Studies-Depression scale and 2 items from the Diagnostic Interview Schedule, with 0.06 as the cutoff point (sensitivity 74%, specificity 87%) (Tuunainen et al., 2001; Wassertheil-Smoller et al., 2004).

### Statistical Analysis

The potential differences in the distribution of baseline characteristics were tested by using the analysis of variance for continuous variables or Chi-square tests for categorical variables across parity categories.

#### Association Between Parity and the Risk of Mild Cognitive Impairment or Probable Dementia

In our study, the incident MCI/dementia was treated as a composite outcome variable, which was commonly used in the pooled analyses of WHIMS trials and several studies (Shumaker et al., 2004; Chen et al., 2015; Tran et al., 2020). Kaplan–Meier survival analysis and Cox proportional hazards regression models were used to evaluate the associations between parity and incident MCI/dementia. The results were presented as hazard ratios (HRs) and 95% confidence intervals (CIs). The proportional hazard assumption was satisfied by analyzing the functions of time through Kaplan–Meier curves and scaled Schoenfeld residuals. The follow-up time for each woman was calculated from the date of WHI randomization (baseline) to the 3MSE examination date, triggering the ultimate classification of MCI or PD (first event of either outcome), death date, or the last date of completing annual cognitive assessment, whichever came first. The following models were applied. Model 1 was adjusted for age, race, and educational level. Model 2 was additionally adjusted for employment status, family income, marital status, social support construct scores, age at menopause, BMI, smoking status, alcohol consumption, physical activities, hypertension, DM, CVD, depression, lipid-lowering medication history, and hormone therapy treatment assignment.

#### Association Between Parity and Cognitive Decline

Linear mixed models with an unstructured variance–covariance structure that was estimated by maximum likelihood were used to estimate the relationships between parity and the change in 3MSE scores over the follow-up period (Roy, 2013). The interaction term of parity (categorical) and time (years since randomization) was included as fixed effects. Moreover, the intercept and slope for time were both fitted as random effects to address intra- and interindividual differences in 3MSE scores over time. We obtained the β-coefficients and 95% CIs from the linear mixed-effects models. A positive β-value for the interaction item of time and parity level indicated that higher parity level was associated with a slower rate of cognitive decline during the study period. Models were adjusted for covariates included in the Cox regression models, except model 2, which was additionally adjusted for baseline 3MSE scores.

#### Subgroup and Sensitivity Analyses

We also stratified the effect estimates by educational levels, prior histories of miscarriage (yes/no), and associated risk factors [obesity, diabetes, hypertension, and depression (yes/no)] (Marden et al., 2017; Xu et al., 2017; Taghdir et al., 2020; Arge et al., 2021; Dol et al., 2021) to assess whether these attributes modified the putative neurocognitive effects of parity using the maximum likelihood ratio test to detect the significance of the interaction effect.

We also conducted the following sensitivity analyses to assess the robustness of our results (Jia et al., 2020). We adopted multiple imputation by chained equations method to impute for participants with key covariates missing at baseline. Baseline characteristics were used to impute the missing values. For each longitudinal analysis, we created 20 imputed data sets and pooled the results using the MI command in Stata (version 15, StataCorp, College Station, TX, United States). The imputation quality was assessed by comparing the imputed data with the original data using density plots (Supplementary Figure 1) (Guo et al., 2021). Restricting analyses were conducted on participants who were 40 years old or younger at menopause (*n* = 585) (Gannon et al., 2018). We used the number of live births as parity instead of term pregnancies (G de Lange et al., 2020). We also examined MCI and PD outcomes separately.

Statistical analyses were performed using Stata (version 15). All analyses were two-sided, and an alpha value of 0.05 was considered the threshold for statistical significance.

## Results

### Distribution of Parity and Population Characteristics

Table 1 presents the population distribution of parity groups in relation to baseline characteristics. In this cohort of 7,100 older women (aged 70.1 ± 3.8 years old), 17.1% were nulliparous, 45.4% had 1–3 term pregnancies, and 37.6% had four or more term pregnancies. Women with higher number of term pregnancies were more likely to be younger, white, married, currently employed, obese, and older at menopause; have a higher level of family income, social support construct scores, and baseline 3MSE scores; and have a history of live/stillbirths, miscarriages, diabetes, and lipid-lowering drug use; they were also less likely to be college graduates (all *P* ≤ 0.017). The baseline characteristics of participants included (*n* = 7,100) or excluded (*n* = 379) from the analysis are compared in Supplementary Table 1.

**TABLE 1:** Baseline characteristic of 7,100 WHIMS participants between parity groups.

Variable	Total*^a^*	Parity groups			*P*-value
		0 (nulliparity)	1–3	≥4 (multiparity)	
*N*	7,100	1,213	3,221	2,666	
Mean follow-up time (years)	10.5 (4.8)	10.2 (5.0)	10.4 (4.7)	10.7 (4.7)	**0.010**
Baseline 3MSE score	95.2 (4.3)	93.8 (5.0)	95.4 (4.1)	95.7 (4.0)	**<0.001**
Age (years)	70.1 (3.8)	70.5 (3.9)	70.3 (3.9)	69.7 (3.7)	**<0.001**
Age at menopause (years)	48.4 (6.5)	47.9 (6.7)	48.4 (6.7)	48.8 (6.3)	**<0.001**
Ethnicity					**<0.001**
White	6,195(87.3)	956 (78.8)	2,863(88.9)	2,376(89.1)	
Non-white	905 (12.8)	257 (21.2)	358 (11.1)	290 (10.9)	
Live births					**<0.001**
Yes	6,511(97.7)	669 (83.6)	3,189(99.4)	2,653(99.9)	
No	152 (2.3)	131 (16.4)	18 (0.6)	3 (0.1)	
Stillbirths					**<0.001**
Yes	355 (5.4)	20 (2.5)	119 (3.8)	216 (8.2)	
No	6,224(94.6)	766 (97.5)	3,050(96.2)	2,408(91.8)	
Miscarriages					**<0.001**
No	4,663(65.7)	1,018(83.9)	2,146(66.6)	1,499(56.2)	
Yes	2,395(33.7)	181 (14.9)	1,059(32.9)	1,155(43.3)	
Missing	42 (0.6)	14 (1.2)	16 (0.5)	12 (0.5)	
Educational level					**<0.001**
<High school	535 (7.5)	100 (8.24)	214 (6.6)	221 (8.3)	
High school/GED	1,572(22.1)	250 (20.6)	662 (20.6)	660 (24.8)	
School after high school	2,836(39.9)	435 (35.9)	1,342(41.7)	1,059(39.7)	
College degree or higher	2,136 30.1)	426 (35.1)	996 (30.9)	714 (26.8)	
Missing	21 (0.3)	2 (0.2)	7 (0.2)	12 (0.5)	
Employment status					**0.002**
Currently employed	1,101(15.1)	169 (13.9)	459 (14.3)	473 (17.7)	
Retired/not working	5,830(82.1)	1,013(83.5)	2,691(83.6)	2,126(79.7)	
Missing	169 (2.4)	31 (2.6)	71 (2.2)	67 (2.5)	
Family income					**<0.001**
≤19,999	1,744(24.6)	324 (26.7)	724 (22.5)	696 (26.1)	
20,000–34,999	2,100(29.6)	343 (28.3)	933 (29.0)	824 (30.9)	
35,000–49,999	1,369(19.3)	217 (17.9)	650 (20.2)	502 (18.8)	
≥50,000	1,463(20.6)	226 (18.6)	734 (22.8)	503 (18.9)	
Missing	424 (6.0)	103 (8.5)	180 (5.6)	141 (5.3)	
Social support construct score*^b^*	35.8 (7.9)	34.8 (8.4)	35.7 (7.9)	36.3 (7.6)	**<0.001**
Marital status					**<0.001**
Married	3,713(52.3)	480 (39.6)	1,742(54.1)	1,491(55.9)	
Not married	3,387(47.7)	733 (60.4)	1,479(45.9)	1,175(44.1)	
Smoking status					0.263
Never smoked	3,718(52.4)	658 (54.3)	1,632(50.7)	1,428(53.6)	
Past smoker	2,787(39.3)	454 (37.4)	1,309(40.6)	1,024(38.4)	
Current smoker	501 (7.1)	85 (7.0)	233 (7.2)	183 (6.9)	
Missing	94 (1.3)	16 (1.3)	47 (1.5)	31 (1.2)	
Alcohol consumption					0.634
Non-drinker	925 (13.0)	167 (13.8)	401 (12.5)	357 (13.4)	
Past drinker	1,395(19.7)	252 (20.8)	624 (19.4)	519 (19.5)	
<1 drink per day	3,922(55.2)	649 (53.5)	1,809(56.2)	1,464(54.9)	
≥1 drink per day	853 (12.0)	143 (11.8)	386 (12.0)	324 (12.2)	
Missing	5 (0.8)	2 (0.2)	1 (0.0)	2 (0.1)	
Physical activity					0.638
No activity	1,269(17.9)	209 (17.2)	563 (17.5)	497 (18.6)	
Some activity	3,209(45.2)	550 (45.3)	1,449(45.0)	1,210(45.4)	
2–4 episodes/week	1,119(15.8)	182 (15.0)	536 (16.6)	401 (15.0)	
≥4 episodes/week	1,496(21.1)	270 (22.3)	670 (20.8)	556 (20.9)	
Missing	7 (0.1)	2 (0.2)	3 (0.1)	2 (0.1)	
BMI (kg/m^2^)					**0.002**
<25	2,058(29.0)	339 (28.0)	1,002(31.1)	717 (26.9)	
25–30	2,576(36.3)	444 (36.6)	1,180(36.6)	952 (35.7)	
≥30	2,425(34.2)	423 (34.9)	1,021(31.7)	981 (36.8)	
Missing	41 (0.6)	7 (0.6)	18 (0.6)	16 (0.6)	
Hypertension					0.112
No	4,262(60.0)	700 (57.7)	1,952(60.6)	1,610(60.4)	
Yes	2,763(38.9)	494 (40.7)	1,234(38.3)	1,035(38.8)	
Missing	75 (1.1)	19 (1.6)	35 (1.1)	21 (0.8)	
Diabetes					**0.004**
No	5,921(83.4)	1,010(83.3)	2,736(84.9)	2,175(81.6)	
Yes	1,167(16.4)	199 (16.4)	483 (15.0)	485 (18.2)	
Missing	12 (0.2)	4 (0.3)	2 (0.1)	6 (0.2)	
CVD					0.123
No	5,772(81.3)	979 (80.7)	2,644(82.1)	2,149(80.6)	
Yes	1,224(17.2)	209 (17.2)	541 (16.8)	474 (17.8)	
Missing	104 (1.5)	25 (2.1)	36 (1.1)	43 (1.6)	
Depression					0.724
No	6,291(88.6)	1,079(89.0)	2,837(88.1)	2,375(89.1)	
Yes	764 (10.8)	125 (10.3)	363 (11.3)	276 (10.4)	
Missing	45 (0.6)	9 (0.7)	21 (0.7)	15 (0.6)	
Lipid-lowering medication history					**<0.001**
No	5,736(80.8)	930 (76.7)	2,589(80.4)	2,217(83.2)	
Yes	1,277(18.0)	268 (22.1)	596 (18.5)	413 (15.5)	
Missing	87 (1.2)	15 (1.2)	36 (1.1)	36 (1.4)	
HT treatment assignment					**0.017**
E-alone control	1,41(20.0)	240 (19.8)	652 (20.2)	525 (19.7)	
E-alone intervention	1,390(19.6)	242 (20.0)	597 (18.5)	551 (20.7)	
E + P control	2,185(30.8)	398 (32.8)	951 (29.5)	836 (31.4)	
E + P intervention	2,108(29.7)	333 (27.5)	1,021(31.7)	754 (28.3)	

*Among participants in each subcategory, differences of categorical variables were examined with the Chi-square test, and continuous variables were examined with analysis of variance. ^a^The total number of subjects summed up across each subcategory varies slightly because of missing values. ^b^The summary score ranges from 9 to 45, where a higher score indicates more social support. WHIMS, the Women’s Health Initiative Memory Study; 3MSE, Modified Mini-Mental State Examination; GED, the Tests of General Educational Development; BMI, body mass index; CVD, cardiovascular disease; HT, hormone therapy; E-alone; conjugated equine estrogen alone; E + P, estrogen plus progestin (medroxyprogesterone acetate). Boldface indicates statistical significance (p < 0.05).*

### Baseline Parity Categories and Risk of Incident Mild Cognitive Impairment/Dementia

**FIGURE 2:**
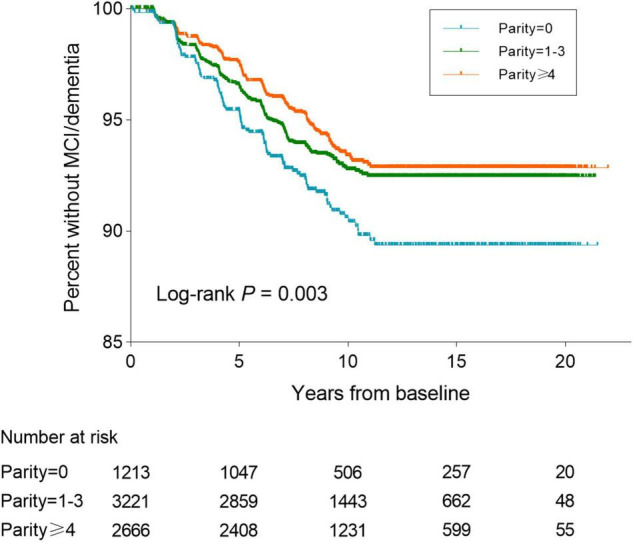
Kaplan–Meier curves of survival to MCI/PD onset over the follow-up stratified by parity groups. MCI, mild cognitive impairment; PD, probable dementia.

During an average of 10.5 years (SD: 4.8) of follow-up, 465 new cases of MCI/dementia (290 MCI and 175 dementia) were identified among 7,100 elder women (Table 2). The Kaplan–Meier curves for unadjusted rates of incident MCI/PD showed differences in risk according to parity categories (Log-rank *P* = 0.003, Figure 2). The numbers of term pregnancies were significantly associated with the risk of MCI/dementia in fully adjusted models (*P* = 0.003). Specifically, compared with nulliparous women, the risk of MCI/dementia was the lowest among multiparous women (HR = 0.71, 95% CI 0.53–0.96), followed by women with 1–3 term pregnancies (HR = 0.75, 95% CI: 0.56–0.99). The rate of MCI/dementia was 6.26 per 1,000 person-years and 8.34, 6.18, and 5.46 per 1,000 person-years for women with 0, 1–3, and 4 or more term pregnancies, respectively.

**TABLE 2:** Hazard ratios and 95% confidence intervals for the associations between parity and the risk of MCI/dementia.

	Person-years	Events/*N*	Event rate (cases per 1,000 person-years)	Unadjusted model		Model 1		Model 2	
				HR (95% CI)	*P*-value	HR (95% CI)	*P*-value	HR (95% CI)	*P*-value
0 (nulliparity)	12343.02	103/1,213	8.34	1 (ref.)		1 (ref.)		1 (ref.)	
1–3	33506.99	207/3,221	6.18	0.73 (0.58–0.93)	**0.010**	0.85 (0.67–1.08)	0.194	0.75 (0.56–0.99)	**0.045**
≥4 (multiparity)	28401.62	155/2,666	5.46	0.65 (0.51–0.84)	**0.001**	0.80 (0.62–1.03)	0.079	0.71 (0.53–0.96)	**0.028**
*P*-value*				**0.004**		0.221		**0.003**	

Model 1: Adjusted for age, race, and educational level. Model 2: Adjusted for age, race, educational level, employment status, family income, marital status, social support construct scores, age at menopause, BMI, smoking status, alcohol consumption, physical activities, hypertension, DM, CVD, depression, lipid-lowering medication history, and hormone therapy treatment assignment. *P-values were from Chi-square tests examining the difference in hazard ratio across parity groups. MCI, mild cognitive impairment; BMI, body mass index; DM, diabetes mellitus; CVD, cardiovascular disease. Boldface indicates statistical significance (p < 0.05).

### Baseline Parity Categories and Cognitive Decline Over Time

Table 3 presents the longitudinal associations between the number of term pregnancies and the rates of change in cognitive function over the first 11 years of the study. Compared with nulliparous women, the rates of global cognitive decline associated with 1 to 3 term pregnancies and multiparity decreased by 0.164 point/year (95% CI: 0.010, 0.319; *P* = 0.037) and 0.292 point/year (95% CI: 0.137, 0.448; *P* < 0.001), respectively, after adjusting for age, race, educational level, employment status, family income, marital status, social support construct scores, age at menopause, BMI, lifestyles, comorbidities, depression, lipid-lowering medication history, hormone therapy treatment, and baseline 3MSE scores.

**TABLE 3:** Estimated mean change in 3MSE score (β coefficients) and 95% confidence intervals by parity groups over the follow-up period.

	Model 1			Model 2		
	β	95% CI	*P*-value	β	95% CI	*P*-value
Time	−0.057	−0.078, −0.036	**<0.001**	−0.061	−0.084, −0.038	**<0.001**
Parity (categorical) × time						
[0 (nulliparity)] × time	ref			ref		
(1–3) × time	0.946	0.716, 1.176	**<0.001**	0.164	0.010, 0.319	**0.037**
[≥4 (multiparity)] × time	1.299	1.067, 1.531	**<0.001**	0.292	0.137, 0.448	**<0.001**

*Model 1: Adjusted for age, race and educational level. Model 2: Adjusted for age, race, educational level, employment status, family income, marital status, social support construct scores, age at menopause, BMI, smoking status, alcohol consumption, physical activities, hypertension, DM, CVD, depression, lipid-lowering medication history, hormone therapy treatment assignment, and baseline 3MSE scores. 3MSE, Modified Mini-Mental State Examination; BMI, body mass index; DM, diabetes mellitus; CVD, cardiovascular disease. Boldface indicates statistical significance (p < 0.05).*

### Additional Analysis

In sensitivity analyses, the observed associations of parity groups with incident MCI/dementia and longitudinal cognitive decline did not materially change, though they were diluted after multiple imputation for missing covariates or exclusion of women who experienced menopause before 40 years old (Supplementary Tables 2–5). When parity was defined as the number of live births, the associations between parity and risk of MCI/dementia and cognitive decline remained significant, and the impact was even enhanced (Supplementary Tables 6, 7). In addition, when examining MCI and PD outcomes separately, similar results showing that parity was associated with lower risk of cognitive impairment were obtained. However, the lowest risk of MCI was observed among multiparous (≥4 term pregnancies) women, whereas the lowest risk of dementia was observed among women with 1–3 pregnancies (Supplementary Tables 8, 9).

**FIGURE 3:**
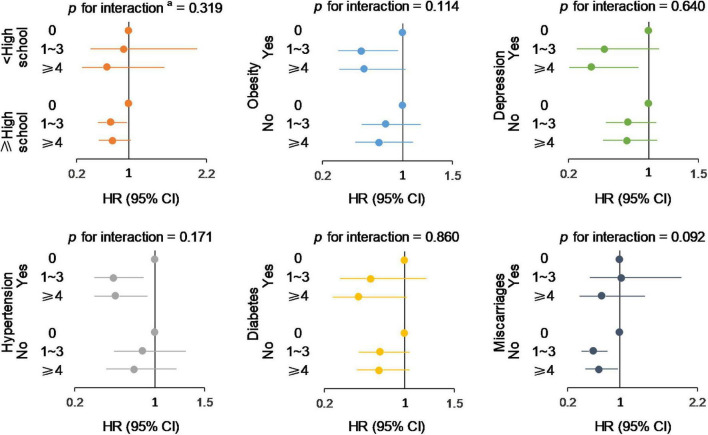
Subgroup analysis of the associations between parity groups and incident MCI/dementia. *^a^*Maximum of likelihood ratios test was used to detect the significance of the interaction effect. Models were adjusted for age, race, educational level, employment status, family income, marital status, social support construct scores, age at menopause, BMI, smoking status, alcohol consumption, physical activities, hypertension, DM, CVD, depression, lipid-lowering medication history, and hormone therapy treatment assignment.

Using the fully adjusted models, we examined whether the association between parity and MCI/dementia was varied by several potential cofounders, including educational level, absence of obesity, depression, hypertension, diabetes, and history of miscarriages (Figure 3). No significant modifying effects were observed for the factors (all *P* for interaction >0.05).

## Discussion

In this large prospective study of 7,100 community-dwelling and cognitively intact older women (≥65 years old), we explored the influence of different levels of parity on the risk of incident MCI/dementia. We further examined the long-term association of the number of parity with cognitive changes. During a follow-up period of 10.5 years on average, we found that higher number of parity was significantly associated with lower risk of MCI/dementia and slower longitudinal rates of cognitive decline. Multiparous (≥4) women showed the lowest risk of MCI/dementia and least cognitive decline compared with nulliparous women, followed by women with 1–3 term pregnancies. These results remained robust after performing several sensitivity and stratified analyses, which made our findings more convincing.

Notably, based on our findings, women with greater number of parity had a lower risk of MCI/dementia, which is in agreement with the findings from a study in the American claims database (Gilsanz et al., 2018). However, the Singapore Chinese Health Study (SCHS) obtained the opposite results (Xingyue et al., 2020), and the Prospective Population Study of Women (PPSW) (Najar et al., 2020) reported no association. Several differences among the studies might explain the results. First, our study and that in the American claims database were conducted in the same country with similar populations (racial and ethnical diversity), whereas SCHS was conducted in Southeast Asia with Chinese populations, and PPSW was conducted in Sweden with Caucasian populations. Second, we used Cox regression models in line with the American claims database and PPSW, whereas SCHS used a multivariable logistic regression. In addition, the statistical models in our study were adjusted for a more extensive, fixed set of covariates that were selected *a priori* from previously published studies (age, race, educational level, employment status, family income, marital status, social support construct scores, age at menopause, BMI, smoking status, alcohol consumption, physical activities, hypertension, DM, CVD, depression, lipid-lowering medication history, and hormone therapy treatment). Third, we defined parity as the number of term pregnancies (lasting six or more months and including live births and stillbirths), whereas the number of parity in PPSW was defined as the sum of children and miscarriages. SCHS merely defined parity as the number of children. The definition of parity in our study presented a more comprehensive way to reflect an individual woman’s lifetime duration of pregnancies by taking into account various conditions of pregnancy history, including live births and stillbirths (lasting 6 or more months), thereby more accurately exploring its effect on cognitive risk (Fox et al., 2013; Gilsanz et al., 2019). Fourth, the American claims database used electronic medical records to identify cognitive outcomes, whereas the other studies used personal health examinations.

More importantly, our findings on cognitive decline in 3MSE added to the growing evidence linking parity with cognitive aging in elderly women. The beneficial effects of higher parity on cognitive function were observed in a study on younger elderly (mean age 57) in Australia (Henderson et al., 2003). Another study on older adults with an average age of 65 in England (Read and Grundy, 2016) also showed that medium parity (two children) was associated with better cognitive functioning compared with low parity (0–1 child), which was comparable with our findings. Furthermore, the slowed decline rates of cognition were observed among women with one or more term pregnancies in the current study; this finding was also comparable with that of another WHI study, in which a higher likelihood of longevity was observed among women with 2–4 term pregnancies (Shadyab et al., 2016). However, not all studies have replicated these findings (Ilango et al., 2019; Jung et al., 2020). Using the Rancho Bernardo Study with participants that were all predominantly white, well educated, and middle class, researchers did not find a statistically significant longitudinal association between parity and cognitive decline (Ilango et al., 2019). In a cross-sectional analysis from Korea (Jung et al., 2020), the grand multiparity group showed lower cognitive scores than the 0–4 parity group, though the relationship was no longer significant after further adjustments. Inconsistencies could be due to the differences in study populations, methodologies, or cognitive scoring methods. This study adds new evidence to support the association of higher parity with slowed decline rates in cognitive functioning, irrespective of the baseline cognitive scores.

Several mechanisms for how reproductive history may play a protective role in cognition during aging have been proposed. Increasing parity is associated with larger gray matter volume (GMV), a well-established biomarker of neuronal aging and AD-related neurodegeneration that often exhibits reduction during the menopause transition and may thus offset the impact of menopause on brain aging in women (Jack et al., 2013; Schelbaum et al., 2021). GMV in brain regions vulnerable to cognitive aging and AD (e.g., temporal clusters) positively correlated with memory and global cognition, thereby suggesting the possible mediation effect of GMV in the association between higher number of parity and reduced risk of MCI/dementia and cognitive decline (Schelbaum et al., 2021). The abovementioned phenomenon could also be explained by the re-emergence of a positive association between high intelligence—as well as other dimensions and correlates of status—and parity in developed countries, particularly high-income countries (Kolk and Barclay, 2019). In present-day affluent societies (high-income countries, e.g., Sweden), higher number of parity seems to be linked with higher income, status, labor force participation, and more resources, thereby playing a pivotal protective role in cognitive decline and dementia (Andersson and Scott, 2007; Sobotka et al., 2011). In addition, increasing number of parity might be related to a later age of menopause and longer duration of reproductive span, all of which are associated with better cognitive performance and delayed cognitive decline (Georgakis et al., 2016; Fu et al., 2021).

The study had several strengths, including the following: relatively large sample; prospective design with long duration of follow-up; geographically, racially, and ethnically diverse population; repeated measures of 3MSE; confirmed and adjudicated MCI or dementia outcomes using standardized criteria; and the availability of information on multiple relevant covariates. Nevertheless, our study had several weaknesses. First, causality in the findings could not be determined owing to the observational nature of the study. However, we deemed that this criticism could be assuaged to some extent, given that all of the participants in this cohort had good cognitive function at enrollment (free of MCI or dementia). Moreover, parity history typically occurred many years before the assessment of cognitive function. Our findings showed that high number of parity was independently associated with a slower longitudinal cognitive decline even after the adjustment of baseline cognitive function, thereby implying that preferable cognition is a corollary of high number of parity but not vice versa. Second, recall bias could not be excluded as the parity data were based on self-reported and retrospective information. However, previous studies have suggested that recall data on reproductive history, including parity, can still be reliable over many years (Must et al., 2002). Thus, self-reported information on parity is widely used in epidemiologic studies. Third, the exclusion of participants with infertility or missing data may be a source of selection bias because their characteristics were different from those of the included participants, and therefore causal inference is limited. Future studies may consider using alternative empirical approaches to gain more traction on this limitation [e.g., polygenic scores for fertility behavior (Barban et al., 2016)]. Fourth, although WHIMS has up to 22 years of follow-up period for survival analyses, the evaluation of cognitive decline over time is limited to the first 10 years in this study. This is due to a transition from face-to-face cognitive evaluations using the 3MSE to a validated telephone cognitive assessment format using the TICS-m. We chose to evaluate cognitive decline over the first 10 years, as this is more proximal to the evaluation of parity. Finally, the 3MSE is a global cognitive function test; therefore, we were unable to pinpoint domain-specific effects. However, it is a widely used cognitive function instrument and easy to administer, making it a strong candidate for cognitive screening in clinical practice (Teng and Chui, 1987).

## Conclusion

This study added new evidence to support the beneficial effects of a high number of parity on the slowing down of cognitive decline in the process of aging among cognitively intact and generally healthy older and postmenopausal women. We found that higher parity was significantly associated with slower decline rates of global cognitive function and subsequent lower risk for developing cognitive impairment or dementia. Future studies are needed to examine the possible mechanisms by which such effects might occur.

## Data Availability Statement

Publicly available datasets were analyzed in this study. This data can be found here: https://www.whi.org/page/whi-memory-study-whims.

## Ethics Statement

The studies involving human participants were reviewed and approved by the National Institutes of Health and Institutional Review Boards. The patients/participants provided their written informed consent to participate in this study.

## Author Contributions

RZ and H-ML wrote the article. RZ, L-WZ, and H-XW performed the data analysis. RZ drafted and critically revised the manuscript. S-YG, M-FC, S-LW, and H-XS provided clinical guidance. L-WZ and X-BW reviewed the language and made substantial interpretation. Y-NH and QZ organized the database. X-BW contributed to the study concept and design, and reviewed the article. All authors contributed to the article and approved the submitted version.

## Conflict of Interest

The authors declare that the research was conducted in the absence of any commercial or financial relationships that could be construed as a potential conflict of interest.

## Publisher’s Note

All claims expressed in this article are solely those of the authors and do not necessarily represent those of their affiliated organizations, or those of the publisher, the editors and the reviewers. Any product that may be evaluated in this article, or claim that may be made by its manufacturer, is not guaranteed or endorsed by the publisher.
