# Supplementary material for: Associations of Parity With Change in Global Cognition and Incident Cognitive Impairment in Older Women
Source: Front Aging Neurosci. 2022 May 4;14:864128. doi: 10.3389/fnagi.2022.864128 (PMC9114765; doi:10.3389/fnagi.2022.864128)

Supplement

Appendix

Women's Health Initiative Memory Study (WHIMS)

The WHIMS was designed to formally assess cognitive function over time among Women's Health Initiative (WHI) participants aged 65 and older. The WHIMS ancillary studies, also based on a subset of participants in the WHI hormone therapy trial, were completed in several phases: during the hormone therapy trial (WHIMS, 1995 through 2002 or 2004, depending on hormone therapy arm), during the post-trial extension period (WHIMS Extension, through 2007), and during the subsequent period (WHIMS Epidemiology of Cognitive Health Outcomes, or WHIMS-ECHO, 2008–present). In the current study, we used data from WHIMS period (1995–2007) and WHIMS-ECHO period (2008–2016). The timeline is shown in Figure A.

Classification of Mild Cognitive Impairment (MCI) and Probable Dementia (PD)

During the WHIMS study period (1995–2007), MCI or PD was ascertained by the validated 4-phase WHIMS protocols [1,2]. Figure B presents a diagram of the phases [1]. In phase 1, all participants completed the Modified Mini-Mental State Exam (3MSE), a test of global cognition, at baseline and annually administered by trained, masked, and certified technicians. Women were screened positively if they scored below a cutoff point on the 3MSE (80 for women with 8 or fewer years of formal education and 88 for those with 9 or more years of formal education) and then proceeded to more extensive neuropsychological testing (phase 2), including a modified Consortium to Establish a Registry for Alzheimer's Disease (CERAD) battery [3]. Each participant and her designated informant were administered a standardized set of 36 items (yes/no) that assessed observed cognitive and behavioral deficits (memory, language, orientation, personality/behavior, basic and instrumental activities of daily living, social and intellectual activities, and judgment and problem solving) [1]. All participants in phase 2 also completed phase 3.

In phase 3, participants subsequently received a detailed clinical neurologic and neuropsychiatric evaluation by clinicians (i.e., neurologists, geriatricians, or geriatric psychiatrists) identified by the local WHIMS clinical center as having experience in diagnosing dementia. WHIMS clinicians were provided with a detailed protocol for the diagnosis. The clinicians reviewed all phase 1 and 2 data on the WHIMS participant

and completed a structured medical history focused on the possible causes of cognitive impairment, as well as physical and neuropsychiatric examinations. The local clinician then classified the WHIMS participant as having no dementia, MCI, or PD based on the Diagnostic and Statistical Manual of Mental Disorders, Fourth Edition (DSM-IV) criteria [4]. MCI was defined as poor performance (≤10th percentile) on at least one CERAD test, evidence of functional impairment (but not severe enough to interfere with activities of daily living), and lack of evidence of psychiatric or other medical disorders (including PD) that could explain the cognitive impairment [5].

Each suspected case of dementia continued to phase 4. They underwent cranial CAT scan and a series of laboratory tests to rule out possible reversible causes of cognitive decline and dementia. If dementia was judged present, the clinician was required to specify the most probable etiology based on all findings. For the diagnosis of PD, the clinician followed the WHIMS protocol based on the DSM-IV criteria. They included detailed descriptions for diagnosis of vascular dementia and Alzheimer's disease, as well as other dementia-related classifications.

Beginning in 2008, the WHIMS-ECHO continued the follow-up of WHIMS. During WHIMS-ECHO (2008–2016), an annual validated cognitive test battery that included the Telephone Interview for Cognitive Status-modified (TICSm) and other tests of memory, language, executive function, and working memory was administered via telephone by certified examiners. A validation study was performed to justify replacing the 3MSE assessment with TICSm, and the results showed that the 3MSE scores predicted by TICSm were highly correlated (0.82) with 3MSE scores [6]. For women who scored below 31 on the TICSm at any annual assessment during the WHIMS-ECHO follow-up, a reliable and pre-identified informant was interviewed via telephone by using the standardized, validated Dementia Questionnaire (DQ) to assess the history of cognitive and behavioral changes, functional impairments, and health events that can affect cognitive functioning [7]. This assessment administered by telephone has been evaluated to be reliable and valid [8]. The results and the cognitive scoring history were then reviewed by a panel of experts in the diagnosis of dementia. A Supplemental Case Ascertainment Protocol (SCAP) was also implemented in the WHIMS-ECHO to identify cases of PD in the deceased and proxy-dependent participants [9]. In the SCAP, the DQ data administrated to a participant-identified proxy and all prior assessments were used for adjudication of dementia.

All clinical and test data of WHIMS and WHIMS-ECHO were then transmitted to a central adjudication committee at the WHIMS Clinical Coordinating Center (CCC; Wake Forest School of Medicine, Winston-Salem, NC, USA), where a panel of experts in diagnosing MCI and dementia independently reviewed cases and made classifications.

Central Adjudication Process for MCI and PD

The central adjudication committee at the WHIMS CCC consists of three board-certified specialists (two neurologists and one geriatric psychiatrist) with extensive experience in diagnosing dementias. The adjudicators independently reviewed all PD cases identified by the local clinician, a random sample of 50% of MCI cases, and a random sample of 10% of cases without dementia. All information on a given participant's test scores, except the field clinician's classification, was provided to two of the three adjudicators, who independently evaluated the data and assigned a classification. The field clinician's diagnostic assessment was then shared with each adjudicator, who independently made a revised diagnosis. If all the adjudicators agreed, this was considered the consensus diagnosis. If they disagreed, the adjudicators discussed the case and attempted to make a consensus classification. The adjudication committee and a geriatric psychologist discussed all cases of disagreement until they reached a consensus classification. The same process was followed to reach consensus on the etiologic classification of the dementia.

Figure A. Timeline.

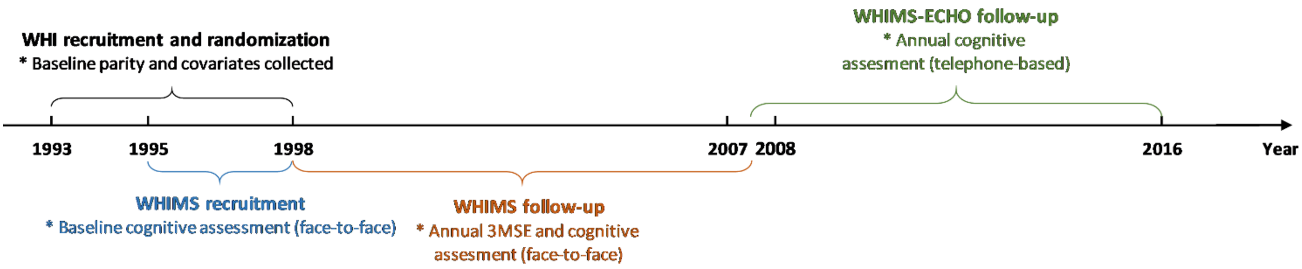

Abbreviations:
WHI: the Women's Health Initiative
WHIMS: the Women's Health Initiative Memory Study
3MSE: the Modified Mini-Mental State Exam
WHIMS-ECHO: the WHIMS Epidemiology of Cognitive Health Outcomes

Figure B. Diagram of the WHIMS study design.

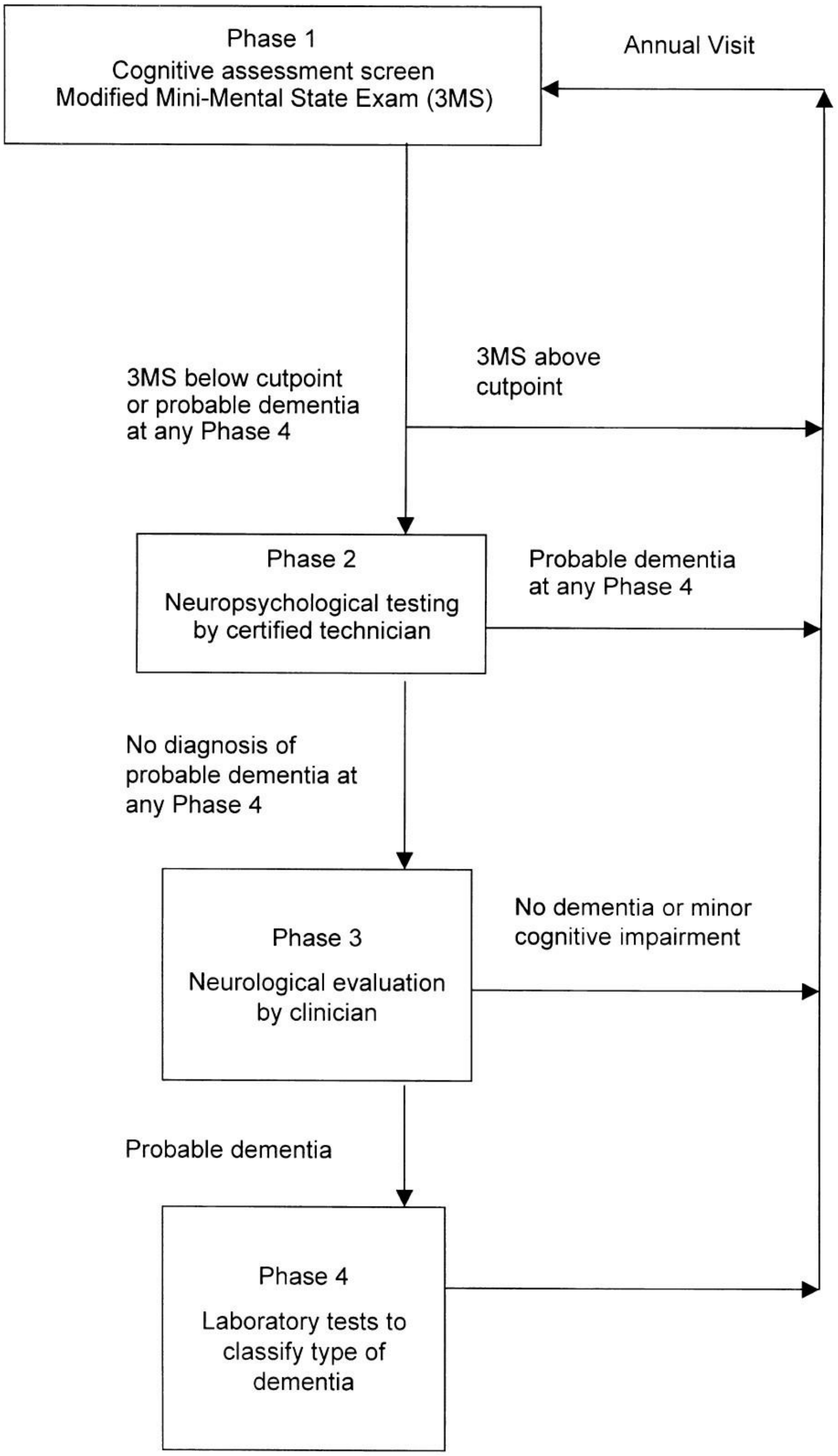
Phase 1
Cognitive assessment screen
Modified Mini-Mental State Exam (3MS)
Annual Visit
3MS below cutpoint
or probable dementia
at any Phase 4
3MS above
cutpoint
Phase 2
Neuropsychological testing
by certified technician
Probable dementia
at any Phase 4
No diagnosis of
probable dementia at
any Phase 4
Phase 3
Neurological evaluation
by clinician
No dementia or minor
cognitive impairment
Probable dementia
Phase 4
Laboratory tests to
classify type of
dementia

Reference:

1 Shumaker S, Reboussin B, Espeland M, *et al.* The Women's Health Initiative Memory Study (WHIMS). *Control Clin Trials* 1998;**19**:604–21. doi:10.1016/S0197-2456(98)00038-5

2 Shumaker S, Legault C, Kuller L, *et al.* Conjugated Equine Estrogens and Incidence of Probable Dementia and Mild Cognitive Impairment in Postmenopausal Women: Women's Health Initiative Memory Study. *JAMA* 2004;**291**:2947–58. doi:10.1001/jama.291.24.2947

3 Morris J, Heyman A, Mohs R, *et al.* The Consortium to Establish a Registry for Alzheimer's Disease (CERAD). Part I. Clinical and neuropsychological assesment of Alzheimer's disease. *Neurology* 1989;**39**:1159–65. doi:10.1212/WNL.39.9.1159

4 American Psychiatric Association. Diagnostic and Statistical Manual of Mental Disorders, 4th ed. (DSM-IV). In: *Diagnostic and Statistical Manual of Mental Disorders: DSM-IV-TR, 4th Edn, text revision*. 2000. doi:10.1176/appi.books.9780890423349

5 Petersen R, Doody R, Kurz A, *et al.* Current Concepts in Mild Cognitive Impairment. *Arch Neurol* 2002;**58**:1985–92. doi:10.1001/archneur.58.12.1985

6 Arnold A, Newman A, Dermond N, *et al.* Using Telephone and Informant Assessments to Estimate Missing Modified Mini-Mental State Exam Scores and Rates of Cognitive Decline. *Neuroepidemiology* 2009;**33**:55–65. doi:10.1159/000215830

7 Kawas C, Segal J, Stewart W, *et al.* A Validation Study of the Dementia Questionnaire. *Arch Neurol* 1994;**51**:901–6. doi:10.1001/archneur.1994.00540210073015

8 Rapp S, Legault C, Espeland M, *et al.* Validation of a Cognitive Assessment Battery Administered over the Telephone. *J Am Geriatr Soc* 2012;**60**:1616–23. doi:10.1111/j.1532-5415.2012.04111.x

9 Gaussoin S, Espeland M, Beavers D, *et al.* Dementia Outcomes after Addition of Proxy-based Assessments for Deceased or Proxy-dependent Participants. *Int J Geriatr Psychiatry* 2019;**34**. doi:10.1002/gps.5130

Supplementary Table 1. Characteristics of the included and excluded participants.

Variable	Included	Excluded	*P* value [a]
N	7,100	379	-
Mean follow-up time (years)	10.5 (4.8)	8.1 (6.0)	**<0.001**
Baseline 3MS score	95.2 (4.3)	94.1 (5.2)	**<0.001**
Age (years)	70.1 (3.8)	69.9 (3.9)	0.22
Age at menopause (years)	48.4 (6.5)	46.8 (7.4)	**<0.001**
Ethnicity			**<0.001**
White	6,195 (87.3)	298 (78.6)	
Non-white	905 (12.8)	81 (21.4)	
Live births			**<0.001**
Yes	6,511 (97.7)	223 (85.8)	
No	152 (2.3)	37 (14.2)	
Still births			0.329
Yes	355 (5.4)	10 (4.0)	
No	6,224 (94.6)	241 (96.0)	
Miscarriages			**<0.001**
No	4,663 (65.7)	280 (73.9)	
Yes	2,395 (33.7)	94 (24.8)	
Missing	42 (0.6)	5 (1.3)	
Educational level			**0.040**
<High school	535 (7.5)	41 (10.8)	
High school/GED	1,572 (22.1)	75 (19.8)	
School after high school	2,836 (39.9)	166 (43.8)	
College degree or higher	2,136 (30.1)	96 (25.3)	
Missing	21 (0.3)	1 (0.3)	
Employment status			0.262
Currently employed	1,101 (15.1)	56 (14.8)	
Retired/not working	5,830 (82.1)	309 (81.5)	
Missing	169 (2.4)	14 (3.7)	
Family income			0.584
≤19,999	1,744 (24.6)	98 (25.9)	
20,000–34,999	2,100 (29.6)	112 (30.0)	
35,000–49,999	1,369 (19.3)	70 (18.5)	
≥50,000	1,463 (20.6)	70 (18.5)	
Missing	424 (6.0)	29 (7.7)	
Social support construct score [b]	35.8 (7.9)	35.2 (8.5)	0.151

Marital status			0.068
Married	3,713 (52.3)	180 (47.5)	
Not married	3,387 (47.7)	199 (52.5)	
Smoking status			**<0.001**
Never smoked	3,718 (52.4)	191 (50.4)	
Past smoker	2,787 (39.3)	143 (37.7)	
Current smoker	501 (7.1)	29 (7.7)	
Missing	94 (1.3)	16 (4.2)	
Alcohol consumption			**0.001**
Non drinker	925 (13.0)	55 (14.5)	
Past drinker	1,395 (19.7)	76 (20.1)	
<1 drink per day	3,922 (55.2)	205 (54.1)	
≥1 drink per day	853 (12.0)	40 (10.6)	
Missing	5 (0.8)	3 (0.8)	
Physical activity			**<0.001**
No activity	1,269 (17.9)	79 (20.8)	
Some activity	3,209 (45.2)	162 (42.7)	
2–4 episodes/week	1,119 (15.8)	56 (14.8)	
≥4 episodes/week	1,496 (21.1)	72 (19.0)	
Missing	7 (0.1)	10 (2.6)	
BMI (kg/m^2)			0.842
<25	2,058 (29.0)	107 (28.2)	
25–30	2,576 (36.3)	132 (34.8)	
≥30	2,425 (34.2)	138 (36.4)	
Missing	41 (0.6)	2 (0.5)	
Hypertension			**0.003**
No	4,262 (60.0)	215 (56.7)	
Yes	2,763 (38.9)	153 (40.4)	
Missing	75 (1.1)	11 (2.9)	
Diabetes			**<0.001**
No	5,921 (83.4)	322 (85.0)	
Yes	1,167 (16.4)	50 (13.2)	
Missing	12 (0.2)	7 (1.9)	
CVD			0.084
No	5,772 (81.3)	305 (80.5)	
Yes	1,224 (17.2)	63 (16.6)	
Missing	104 (1.5)	11 (2.9)	

Depression			0.077
No	6,291 (88.6)	329 (86.8)	
Yes	764 (10.8)	44 (11.6)	
Missing	45 (0.6)	6 (1.6)	
Lipid-lowering medication history			**<0.001**
No	5,736 (80.8)	297 (78.4)	
Yes	1,277 (18.0)	68 (17.9)	
Missing	87 (1.2)	14 (3.7)	
HT treatment assignment			0.720
E-alone control	1,41 (20.0)	67 (17.7)	
E-alone intervention	1,390 (19.6)	79 (20.8)	
E+P control	2,185 (30.8)	117 (30.9)	
E+P intervention	2,108 (29.7)	116 (30.6)	

Values are presented as the mean ± SD for continuous variables and number (%) for categorical variables.

[a] *P* values were derived using the Chi-square test for categorical variables and the t-test for continuous variables.

Abbreviations: 3MS, Modified Mini-Mental State Examination; GED, the Tests of General Educational Development; BMI, body mass index; CVD, cardiovascular disease; HT, hormone therapy; E-alone; conjugated equine estrogen alone; E+P, estrogen plus progestin (medroxyprogesterone acetate).

Supplementary Table 2. Hazard ratios and 95% confidence intervals for the association between parity and the risk of MCI/dementia after multiple imputations.

	HR (95% CI)	*P* value
0 (nulliparity)	1 (ref.)	
1–3	0.80 (0.61–1.04)	0.091
≥4 (multiparity)	0.75 (0.57–0.99)	**0.040**

Model was adjusted for age, race, educational level, employment status, family income, marital status, social support construct scores, age at menopause, BMI, smoking status, alcohol consumption, physical activities, hypertension, DM, CVD, depression, lipid-lowering medication history, and hormone therapy treatment assignment.

* *P* values were from Chi-square tests examining the difference in hazard ratio across parity groups.

Abbreviations: MCI, mild cognitive impairment; BMI, body mass index; DM, diabetes mellitus; CVD, cardiovascular disease.

Only model 2 was presented.

Supplementary Table 3. Estimated mean change in 3MS score (β-coefficients) and 95% confidence intervals by parity groups over the follow-up after multiple imputations (n=7,100).

	β	95% CI	*P* value
Time	-0.066	-0.088, -0.044	**<0.001**
Parity (categorical) × time			
(0 [nulliparity]) × time	ref		
(1–3) × time	0.160	0.016, 0.305	**0.030**
(≥4 [multiparity]) × time	0.300	0.154, 0.445	**<0.001**

Model was adjusted for age, race, educational level, employment status, family income, marital status, social support construct scores, age at menopause, BMI, smoking status, alcohol consumption, physical activities, hypertension, DM, CVD, depression, lipid-lowering medication history, hormone therapy treatment assignment, and baseline 3MSE scores.

Abbreviations: 3MS, Modified Mini-Mental State Examination; BMI, body mass index; DM, diabetes mellitus; CVD, cardiovascular disease.

Only model 2 was presented.

Supplementary Table 4. Hazard ratios and 95% confidence intervals for the associations between parity and the risk of MCI/dementia after excluding women who experienced menopause before 40 years old (n=6,515).

	Person-years	Events/N	Event rate (cases per 1000 person-years)	Unadjusted model		Model 1		Model 2	
				HR (95% CI)	*P* value	HR (95% CI)	*P* value	HR (95% CI)	*P* value
0 (nulliparity)	11211.63	91/1,101	8.57	1 (ref.)		1 (ref.)		1 (ref.)	
1–3	30686.32	186/2,935	4.99	0.74 (0.57–0.95)	**0.018**	0.86 (0.67–1.11)	0.249	0.76 (0.56–1.03)	0.074
≥4 (multiparity)	26521.15	141/2,479	5.59	0.65 (0.50–0.85)	**0.002**	0.80 (0.62–1.05)	0.103	0.70 (0.51–0.97)	**0.031**
P value*				**0.008**		0.226		**0.007**	

Model 1: adjusted for age, race, and educational level.

Model 2: adjusted for age, race, educational level, employment status, family income, marital status, social support construct scores, age at menopause, BMI, smoking status, alcohol consumption, physical activities, hypertension, DM, CVD, depression, lipid-lowering medication history, and hormone therapy treatment assignment.

* *P* values were from Chi-square tests examining the difference in hazard ratio across parity groups.

Abbreviations: MCI, mild cognitive impairment; BMI, body mass index; DM, diabetes mellitus; CVD, cardiovascular disease.

Supplementary Table 5. Estimated mean change in 3MS score (β-coefficients) and 95% confidence intervals by parity groups over the follow-up after excluding women who experienced menopause before 40 years old (n=6,515).

	Model 1			Model 2		
	β	95% CI	*P* value	β	95% CI	*P* value
Time	-0.058	-0.080, -0.037	**<0.001**	-0.063	-0.086, -0.039	**<0.001**
Parity (categorical) × time						
(0 [nulliparity]) × time	ref			ref		
(1–3) × time	0.907	0.664, 1.148	**<0.001**	0.153	-0.009, 0.315	0.065
(≥4 [multiparity]) × time	1.301	1.059, 1.544	**<0.001**	0.296	0.133, 0.459	**<0.001**

Model 1: adjusted for age, race, and educational level.

Model 2: adjusted for age, race, educational level, employment status, family income, marital status, social support construct scores, age at menopause, BMI, smoking status, alcohol consumption, physical activities, hypertension, DM, CVD, depression, lipid-lowering medication history, hormone therapy treatment assignment, and baseline 3MSE scores.

Abbreviations: 3MS, Modified Mini-Mental State Examination; BMI, body mass index; DM, diabetes mellitus; CVD, cardiovascular disease.

Supplementary Table 6. Hazard ratios and 95% confidence intervals for the associations between parity (defined as the number of live births) and the risk of MCI/dementia (n=7,191).

	Person-years	Events/N	Event rate (cases per 1000 person-years)	Unadjusted model		Model 1		Model 2	
				HR (95% CI)	*P* value	HR (95% CI)	*P* value	HR (95% CI)	*P* value
0 (nulliparity)	12560.06	107/1,234	8.52	1 (ref.)		1 (ref.)		1 (ref.)	
1–3	34313.68	210/3,294	6.12	0.71 (0.56–0.90)	**0.004**	0.82 (0.65–1.04)	0.097	0.71 (0.54–0.94)	**0.016**
≥4 (multiparity)	28301.36	158/2,663	5.58	0.65 (0.51–0.83)	**0.001**	0.79 (0.61–1.01)	0.062	0.69 (0.51–0.97)	**0.016**
P value*				**0.003**		0.167		**0.002**	

Model 1: adjusted for age, race, and educational level.

Model 2: adjusted for age, race, educational level, employment status, family income, marital status, social support construct scores, age at menopause, BMI, smoking status, alcohol consumption, physical activities, hypertension, DM, CVD, depression, lipid-lowering medication history, and hormone therapy treatment assignment.

* *P* values were from Chi-square tests examining the difference in hazard ratio across parity groups.

Abbreviations: MCI, mild cognitive impairment; BMI, body mass index; DM, diabetes mellitus; CVD, cardiovascular disease.

Supplementary Table 7. Estimated mean change in 3MS score (β-coefficients) and 95% confidence intervals by parity groups (defined as the number of live births) over the follow-up (n=7,191).

	Model 1			Model 2		
	β	95% CI	*P* value	β	95% CI	*P* value
Time	-0.057	-0.077, -0.036	**<0.001**	-0.061	-0.084, -0.038	**<0.001**
Parity (categorical) × time						
(0 [nulliparity]) × time	ref			ref		
(1–3) × time	0.957	0.728, 1.186	**<0.001**	0.193	0.041, 0.346	**0.013**
(≥4 [multiparity]) × time	1.284	1.052, 1.516	**<0.001**	0.320	0.166, 0.474	**<0.001**

Model 1: adjusted for age, race, and educational level.

Model 2: adjusted for age, race, educational level, employment status, family income, marital status, social support construct scores, age at menopause, BMI, smoking status, alcohol consumption, physical activities, hypertension, DM, CVD, depression, lipid-lowering medication history, hormone therapy treatment assignment, and baseline 3MSE scores.

Abbreviations: 3MS, Modified Mini-Mental State Examination; BMI, body mass index; DM, diabetes mellitus; CVD, cardiovascular disease.

Supplementary Table 8. Hazard ratios and 95% confidence intervals for the associations between parity and the risk of MCI (n=6,925).

	Person-years	Events/N	Event rate (cases per 1000 person-years)	Unadjusted model		Model 1		Model 2	
				HR (95% CI)	*P* value	HR (95% CI)	*P* value	HR (95% CI)	*P* value
0 (nulliparity)	12143.82	62/1,172	5.11	1 (ref.)		1 (ref.)		1 (ref.)	
1–3	33147.82	137/3,151	4.13	0.80 (0.59–1.08)	0.145	0.97 (0.72–1.32)	0.846	0.84 (0.59–1.19)	0.322
≥4 (multiparity)	28027.46	91/2,602	3.25	0.63 (0.46–0.88)	**0.006**	0.79 (0.57–1.10)	0.164	0.64 (0.43–0.94)	**0.021**
P value[a]				**0.021**		0.203		**0.022**	
P for trend[b]				**0.006**		0.135		**0.022**	

Model 1: adjusted for age, race, and educational level.

Model 2: adjusted for age, race, educational level, employment status, family income, marital status, social support construct scores, age at menopause, BMI, smoking status, alcohol consumption, physical activities, hypertension, DM, CVD, depression, lipid-lowering medication history, and hormone therapy treatment assignment.

[a] *P* values were from Chi-square tests examining the difference in hazard ratio across parity groups.

[b] *P* for trend was tested by treating parity groups as continuous variable.

Abbreviations: MCI, mild cognitive impairment; BMI, body mass index; DM, diabetes mellitus; CVD, cardiovascular disease.

Supplementary Table 9. Hazard ratios and 95% confidence intervals for the associations between parity and the risk of dementia (n=6,810).

	Person-years	Events/N	Event rate (cases per 1000 person-years)	Unadjusted model		Model 1		Model 2	
				HR (95% CI)	*P* value	HR (95% CI)	*P* value	HR (95% CI)	*P* value
0 (nulliparity)	12025.69	41/1,151	3.41	1 (ref.)		1 (ref.)		1 (ref.)	
1–3	32864.32	70/3,084	2.13	0.62 (0.42–0.90)	**0.014**	0.68 (0.46–1.00)	0.051	0.59 (0.37–0.93)	**0.023**
≥4 (multiparity)	27929.12	64/2,575	2.29	0.67 (0.45–0.99)	**0.044**	0.80 (0.53–1.19)	0.265	0.71 (0.44–1.15)	0.163
P value[a]				**0.048**		0.102		**0.026**	
P for trend[b]				0.104		0.423		0.111	

Model 1: adjusted for age, race, and educational level.

Model 2: adjusted for age, race, educational level, employment status, family income, marital status, social support construct scores, age at menopause, BMI, smoking status, alcohol consumption, physical activities, hypertension, DM, CVD, depression, lipid-lowering medication history, and hormone therapy treatment assignment.

[a] *P* values were from Chi-square tests examining the difference in hazard ratio across parity groups.

Abbreviations: BMI, body mass index; DM, diabetes mellitus; CVD, cardiovascular disease.

Supplementary Figure 1. Density plots of comparison between imputed and original data. The red lines represent the imputed data, whereas the blue line represents the original data.

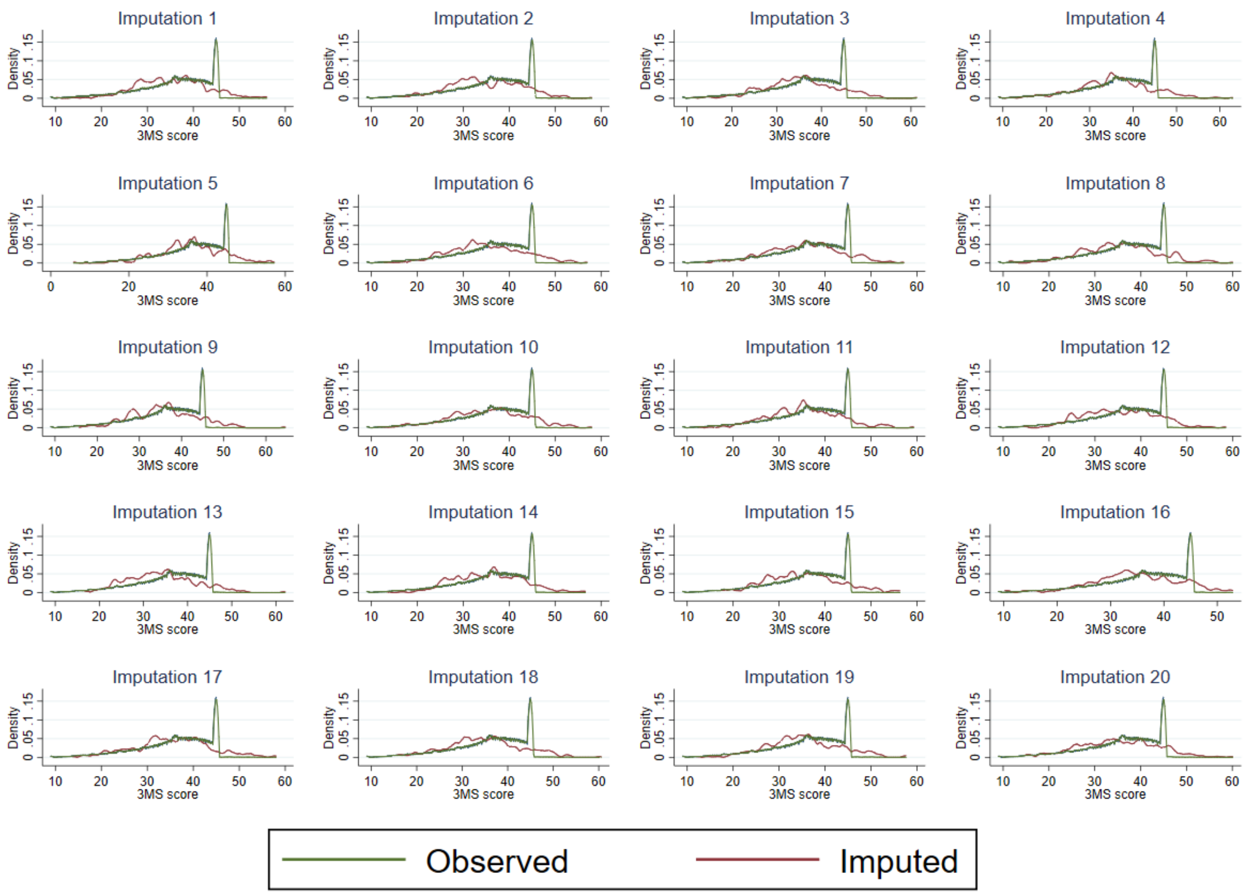

Supplement: Supplementary file 1 [file Data_Sheet_1.pdf]
